# Decision making process in simultaneous laparoscopic resection of colorectal cancer and liver metastases. Review of literature^1^

**DOI:** 10.1590/s0102-865020200030000008

**Published:** 2020-05-22

**Authors:** Raphael Leonardo Cunha de Araujo, Marleny Novaes Figueiredo, Marco Aurélio de Sanctis, Luís Gustavo Capochin Romagnolo, Marcelo Moura Linhares, Armando Geraldo Franchini Melani, Jacques Marescaux

**Affiliations:** I PhD, Department of Upper GI and HPB Surgery, Hospital do Câncer de Barretos; Department of Digestive Surgery, Universidade Federal de São Paulo (UNIFESP), and Hospital Israelita Albert Einstein, Sao Paulo-SP, Brazil. Conception and design of the study.; II PhD, Hospital Municipal da Vila Santa Catarina, and Universidade Nove de Julho (UNINOVE), Sao Paulo-SP, Brazil. Conception and design of the study. Conception and design of the study.; III MD, Department of Upper GI and HPB Surgery, Hospital do Câncer de Barretos, Brazil. Illustrations.; IV MD, Research Institute against Cancer of the Digestive System, Strasbourg, France, and Department of Oncologic Colorectal Surgery, Hospital do Câncer de Barretos, Brazil. Manuscript writing, critical revision.; Hospital do Câncer de Barretos, Department of Oncologic Colorectal Surgery, Brazil; V PhD, Department of Digestive Surgery, UNIFESP, Sao Paulo-SP, Brazil. Manuscript writing, critical revision.; VI MD, MSc, Research Institute against Cancer of the Digestive System, Strasbourg, France. Manuscript writing, critical revision.; VII MD, Research Institute against Cancer of the Digestive System, Strasbourg, France. Manuscript writing, critical revision.

**Keywords:** Colorectal Neoplasms, Neoplasms, Multiple Primary, Laparoscopy, Hepatectomy, Colectomy

## Abstract

**Purpose:**

The benefits of laparoscopic approaches to treat colorectal cancer (CRC) and colorectal liver metastases (CRLM) separately are well established. However, there is no consensus about the optimal timing to approach the primary tumor and CRLM, whether simultaneously or staged. The objective of this review with practical reports is to discuss technical aspects required for patient selection to perform simultaneous laparoscopic approaches for CRC and CRLM.

**Methods:**

Literature review of oncological factors associated with patient selection for surgical treatment of CRLM and the use of laparoscopy in those cases, and report of technical aspects for simultaneous CRC and CRLM approaches.

**Results:**

Simultaneous laparoscopic resection has been successful in many series of selected patients, although it seems to be safer to perform minor and major liver resection with non-extended colorectal resections, and to avoid two high-risk procedures at the same time.

**Conclusions:**

Simultaneous CRC and CRLM resections seem to be safe when patients are carefully selected, also considering the risk of recurrence concerning oncologic outcomes. The pre-planning of simultaneous resection is mandatory to plan trocar positioning, procedure sequencing, and patient position.

## Introduction

Colorectal cancer (CRC) is the third most common malignancy worldwide with 1.849,518 new cases estimated for 2018, and it represents the third most common malignancy in men (23.6%) and second in women (16.3%), and the forth (10.8%) and third (7.2%) cause of death, respectively^1^. In the United States, CRC represents the third most common malignancy for both men and women; with 75.610 and 64.640 new cases estimated for 2018, respectively and it represents 8% of estimated deaths; with 27.390 and 23.240 estimated deaths respectively^2^. Colorectal liver metastases (CRLM) are present in 60% of patients with colorectal cancer during the course of the disease and 15–20% of patients present CRLM at the time of diagnosis^3^. Furthermore, the presence of synchronous CRLM is well established as a prognostic factor for recurrence and death^4^ ^–^ ^6^.

The laparoscopic approach for CRLM is also a valid option to treat both CRC and CRLM with an increasing acceptance in the surgical practice^7^ ^,^ ^8^. Moreover, the surgical timing for synchronous CRLM, with either simultaneous or staged resection, does not seem to impact the recurrence-free survival (RFS) or the overall survival (OS)^9^ ^,^ ^10^. It is noteworthy that to the best of our knowledge, there are no randomized clinical trials comparing the results of simultaneous or staged surgery for colorectal synchronic tumors with liver metastases, by open or laparoscopic surgery. The objective of this review is to highlight and discuss the technical aspects required to select and perform simultaneous laparoscopic surgery for the primary tumor and the CRLM.

## Oncological patient selection

Patient selection is key to obtain the best clinical and oncological results after simultaneous colorectal cancer and liver metastases resection. The identification of patients at a low risk of recurrent disease is probably the most important feature to predict good oncological results. The presence of synchronous CRLM represents an important prognostic factor for RFS and OS. The definition of synchronous metastases in the literature varies and includes metastases at the time of diagnosis or even before the diagnosis of the primary site, and also metastases discovered 6 or 12 months after the time of diagnosis, varying according to author's definitions from each study^5^ ^,^ ^11^. Fong *et al*.^5^ described a clinical risk score (CRS) that includes the presence of synchronous disease as an independent prognostic factor, which predicts the risk of recurrence after curative-intent liver resection of CRLM in 1.001 patients. It has become one of the most commonly used CRSs since the criteria can be obtained from the pre-operative evaluation based on the pathologic stage of the primary tumor, imaging workup and carcinoembryonic antigen (CEA) level. This CRS represents a low-cost preoperative evaluation since both pathology and pre-operative imaging are always necessary for pre-operative staging and surgical planning. Although CEA is not strictly necessary for diagnosis, its baseline expression is important to identify those patients that express CEA and it is used as a surrogate for tumor burden, and also for monitoring and predicting postoperative recurrence, even for disease undetectable on radiological imaging^12^. Fong's CRS criteria include positive nodal status of the primary tumor, disease-free interval (DFI – from primary to CRLM) < 12 months, number of liver lesions >1, preoperative carcinoembryonic antigen (CEA) level >200 ng/ml, and size of the largest tumor >5 cm^5^. One point was assigned for each criterion with a score ranging from 0 to 5, which represents 5-y OS rates of 60% and 74 months for score 0, down to 22% and 14 months for score 5. For the purpose of this review, patients with scores of 0, 1, or 2 were considered as low-risk of recurrence, and patients with scores of 3, 4 or 5 were considered as having a high risk of recurrence. The specific population of synchronous CRLM represents a certain risk of recurrence, since it already counts one point on CRS, even with the use of modern chemotherapy regimens^4^ ^,^ ^13^.

Concerning the use of molecular markers, they can also provide more information to identify patients with a higher risk of recurrence. Brudvik *et al*.^14^ reported in a systematic review with meta-analysis of retrospective studies data showing that KRAS mutations were associated with both lower RFS (HR: 1.89) and lower OS (HR: 2.23). BRAF mutation was also identified as an independent prognostic factor of recurrence associated with a worse survival (HR 3.90)^15^. These data should be seen with parsimony, as a hypothesis generator, since they were derived from retrospective data. Guinney *et al*.^16^ reported a consensus about molecular subtypes of colorectal cancer proposing a molecular classification with at least four CRC subtypes associated with different prognoses: CMS1 (microsatellite instability and immune activation features, better prognosis), CMS2 (epithelial, with marked WNT, and MYC signaling activation), CMS3 (metabolic dysregulation), and CMS4 (mesenchymal features, worse outcome). Although the use of molecular markers to select patients for liver resections, upfront or after chemotherapy, seems to be a future direction, both retrospective and prospective validations of this molecular classification in patients undergoing curative-intent resection of CRLM (R0), with and without additional chemotherapy, is crucial for a proper identification of patients at risk^17^.

The timing of surgery for CRC and CRLM does not seem to influence oncologic outcomes. Brouquet *et al*.^9^ reported a series of 142 patients with initially resectable synchronous CRLM and primary tumor who underwent curative-intent treatment using three different approaches: 72 patients underwent the classic approach (primary tumor before liver), 43 underwent a combined strategy (simultaneous resection of primary tumor and liver), and 27 received a reverse approach (liver before primary)^9^. No differences in postoperative mortality rates, postoperative cumulative morbidity rates, and 3- and 5-year OS rates in the combined, classic and reverse strategy groups were observed. Nonetheless, a selection bias was detected based on the median number of CRLM per patient: 1 in the combined group, 3 in the classic group, and 4 in the reverse strategy group (P=0.01 classic vs. reverse; P < 0.001 reverse vs. combined). Thus, it seems that the liver tumor burden and presence of symptoms for the primary tumor play a significant role in the choice of reverse, combined and classic strategies and all of them are possible to promote a curative-intent treatment^9^ ^,^ ^17^. Moreover, Silberhumer *et al*.^10^ ^,^ ^18^ published two series showing neither harm nor benefit for both surgical and oncologic outcomes in patients undergoing simultaneous resection of rectal cancer and CRLM. Different results are observed in the data analysis of the European base LiverMetSurvey, with more than 16,000 cases of liver metastases of colorectal origin and 3.144 cases of synchronous tumors^11^. The authors showed a significantly lower 5-y OS in the simultaneous resection group than in the liver first surgery and primary-first surgery (40%, 47% and 44%, respectively), but different patient selection criteria for the 3 groups cannot be ruled out in this multicenter database. Therefore, the influence of surgical timing on oncological outcomes for colorectal synchronic disease still presents conflicting results and it will only be determined in a future randomized clinical trial.

**Figure 1:**
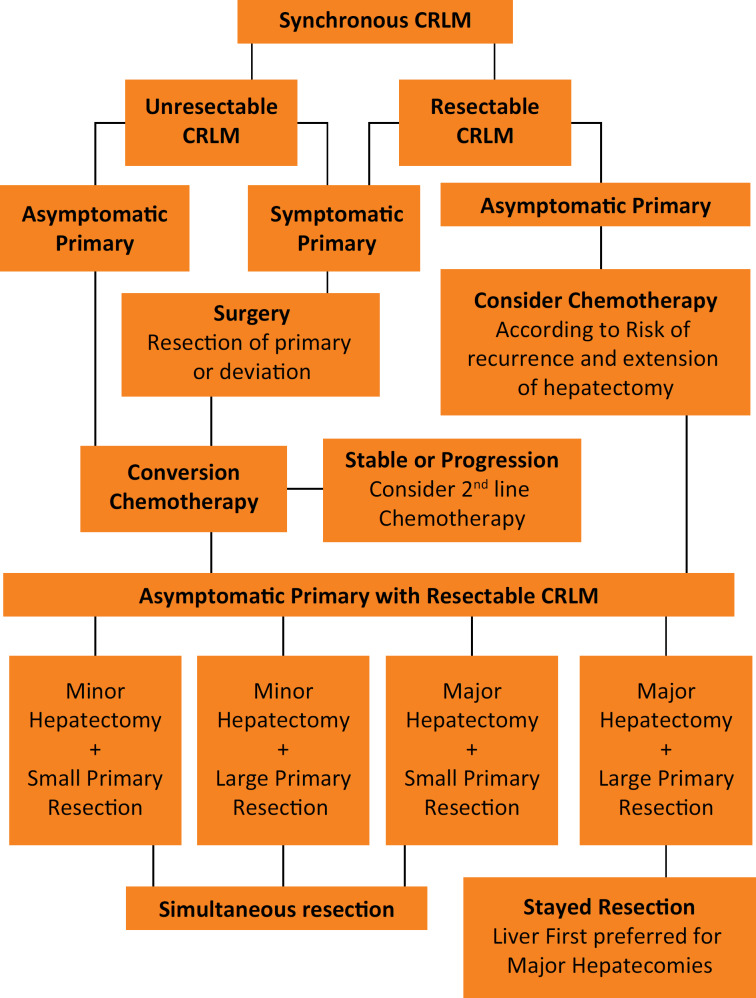
This flowchart represents the decision-making process for the simultaneous surgical treatment of resectable colorectal liver metastases based on clinical presentation and its risk of complications and recurrence. (CRLM – Colorectal Liver Metastases)

Another patient selection instrument is the use of chemotherapy for those patients with asymptomatic disease. The use of associated systemic chemotherapy in patients undergoing curative-intent resection has improved RFS rates in randomized clinical trials (RCTs) and increased OS in retrospective series and meta-analysis^19^ ^–^ ^22^. Whether the chemotherapy should be given before and after or only after liver surgery remains an open debate and there is no RCT on this issue. A series of 411 patients with initially resectable CRLM was reported and demonstrated how patient selection was performed in ten years of practice at Memorial Sloan-Kettering Cancer Center^13^. No differences in OS were detected, but the RFS rates were significantly better for those who received adjuvant chemotherapy than for patients in the perioperative regimen group (5-year RFS of 38% and 31%, respectively, p=0.036). However, the differences disappeared once the RFS was adjusted for CRS (high and low risk of recurrence according to Fong's CRS), and the difference between groups was no longer statistically significant. Nowadays, the use of chemotherapy works both as a selection tool for “good responders” and conversion therapy and its use should be included in the patient selection process, as demonstrated in Figure 1. Briefly, patients with high risk of recurrence, high risk of postoperative liver failure from putative small remnant liver volume, should not be operated upfront since the tumor biology and its chemo response can be tested by optimizing patient selection and eventually result in CRLM downsizing for “good responders”, or occasionally to avoid an unnecessary surgery in “bad responders”, e.g. patients who progress during chemotherapy.

Regardless of the primary site being in the rectum or the colon, the presence of symptoms is one the most important factors in the management of these patients, and it works similarly for both rectal and colon cancer, regarding need of deviation, colonic stent or resection upfront. Symptomatic patients must be operated (oncologic resection when possible, or deviation with colostomy or colonic stents) according to their symptoms (generally bleeding or obstruction) before the definitive treatment of the primary tumor and the CRLM. The lack of RCTs addressing simultaneous or staged resections for synchronous CRC and CRLM is an important limitation for a more precise patient selection in open or minimally invasive surgery. It represents a limitation for the decision-making process using evidence-based medicine and is consequently an important limitation of this study as well.

## Surgical planning

Although the rationale of the timing of resection (liver first, colon first or simultaneous resection) does not seem to impact the overall survival, many considerations should be taken into account regarding the surgical planning to avoid surgical complications^9^ ^,^ ^23^. Feng *et al*.^23^ demonstrated in a systemic review and meta-analysis of non-randomized trials and retrospective series that there is no impact on OS when simultaneous and staged resections are compared. This meta-analysis also showed no impact in surgical outcomes; however, an important analysis of imbalance showed a remarkable patient selection for simultaneous resection based on a smaller number of lesions, smaller size of lesions, preferred unilobar disease and preferred right colon tumor location instead of the rectum. This undeniable selection bias appears to report the real clinical practice, which avoids adding two surgeries with a high risk of complications, as demonstrated in Table 1. There was a selection bias to perform simultaneous resection in patients with less tumor burden and more favorable laparoscopic liver resection.

**Table 1:** Comparison of surgical outcomes between laparoscopic and open simultaneous resection of colorectal liver metastases

Author	Year	n		Age Mean/Median		Operative time - minutes Mean/Median		Blood loss – mL Mean/Median		Morbidity (%)		Hospital stay – days Mean/Median	
		Lap	Open	Lap	Open	Lap	Open	Lap	Open	Lap	Open	Lap	Open
Huh^37^	2010	20	20	63 (36-71)	62 (44-85)	358 (215-595)	278 (140-465)	350 (120-950)	500 (100-1200)	10 (50)	8 (40)	10 (7-30)	10 (7-31)
Chen^38^	2011	23	18	.	.	350	342	275	590	34.8%	50%	12	16
Hu^39^	2012	13	13	54 ± 10	53 ± 11	313 ± 44	350 ± 46	258 ± 111	273 ± 95	1 (7)	0	8.5 ± 1.9	11.2 ± 1.8
Takasu^40^	2013	7	7	74 ± 12	62 ± 5	472 ± 90	466 ± 107	152 ± 128	496 ± 191	12.5%	33.3%	16.2 ± 6.1	36.1 ± 24.9
Jung^41^	2014	24	24	60 (43-75)	60 (37-0)	290 (183-551)	244 (149-375)	325 (50-900)	250 (50-850)	4 (17)	10 (42)	8 (5-23)	10.5 (8-23)
Lin^42^	2014	36	36	57.5 ± 7.3	57.4 ± 10.4	317.5 ± 47.4	251.7+-49.6	278.1 ± 173.3	382.5 ± 145.6	9 (25)	11 (30.5)	7.4 ± 1.6	9 ± 3.5
Tranchart^43^	2015	89	89	66.6 ± 10.8	65 ± 9.4	332 ± 110	308 ± 86	229 ± 228	188 ± 207	13 (15)	13 (15)	10.3 ± 9.6	12.2 ± 9.2
Ratti^44^	2016	25	25	.	.	420	310	350	600	64%	66%	7	9
Xu^45^	2017	20	20	58.2 ± 10.6	59.6 ± 10.8	246.7 ± 78.2	248.3 ± 79.9	175 (100-275)	300 (162.5-575)**	3 (15)	5 (25)	9 (8-11)	12 (10-16)
Ivanecz^46^	2018	10	10	62.2 ± 7.9	65.4 ± 8.1	261 ± 92.8	257 ± 66.8	105 (30-180)	170 (70-230)***	5 (50)	3 (30)	8 (8-12)	11.5 (10-33)

Open simultaneous resection for colorectal cancer and synchronic liver metastasis has been established as a safe approach with good surgical outcomes, even when major hepatectomies are performed, and also when the rectum is the site of the primary tumor^9^ ^,^ ^18^ ^,^ ^24^ ^,^ ^25^. Laparoscopic simultaneous resections have also been attempted with success in case reports and small series^26^ ^–^ ^30^. Ferretti *et al*.^31^ were the first group to report results of laparoscopic simultaneous resection in a large series of patients (n=142), as a multicenter international study including 14 experienced centers worldwide. Only 7 patients required conversion to open surgery and the overall morbidity rate was 31%. Liver-related morbidity was 7.7%. Overall 1-, 3- and 5-year survival was 98.8, 82.1 and 71.9% respectively, while DFS was 85.6, 65.9 and 63%.

Thus, based on non-randomized data, it seems to be safer to perform both minor and major liver resections with small colorectal resections, but major hepatectomy and enlarged colorectal resections should not be approached at the same time to avoid increasing complications, as suggested in Figure 1. For both high-risk procedures, the liver first approach could be considered if it represents an extensive resection, avoiding occasional progression to unresectable status.

## Intraoperative course

**Figure 2:**
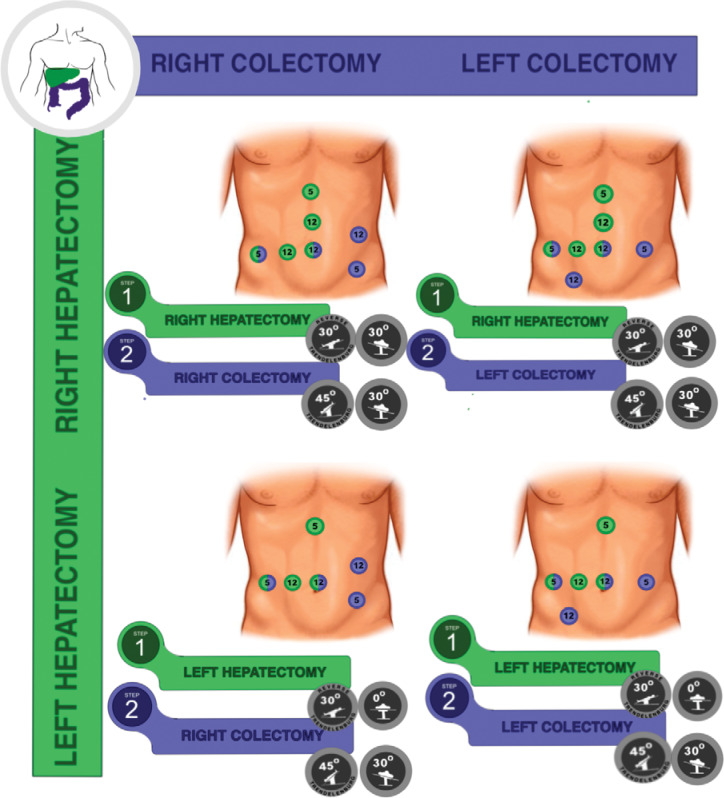
The position of the patient according to sequence and site of surgeries.

Regarding the intraoperative course, the trocar positions are usually different for the liver and colorectal approaches. Most of the time, it requires an adaptation of surgical teams to share some trocars. Since there is no consensus in the literature about this specific issue, we report our experience in the Barretos Cancer Hospital according to the location of both primary and CRLM, adapted to major hepatectomies, as demonstrated in Figure 2. The position of the patient should also change according to the sequence and site of surgeries, as reported in Figure 2. The proposed rational is to adapt the trocar positioning to optimize the smaller number of trocars in liver and colorectal resections.

The use of low central venous pressure (CVP) is usually requested since it decreases the risk of intraoperative bleeding^32^. The rationale is to avoid bleeding from suprahepatic veins because they do not present valves, thus CVP represents exactly the pressure inside of veins favoring hepatic bleeding. The main issue with low CVP is dehydration, since an important fluid restriction is necessary, and low renal flow that may cause transient pre-renal failure^32^. Thus, in our surgical practice, we prefer to start the procedure by the liver maintaining a low CVP and use more fluid intake during the colorectal resection to recover renal balance without a higher risk of hepatic bleeding. Another strategy usually adopted in liver surgery is the hilar pedicle clamping, also called Pringle Maneuver, to control inflow during hepatectomy^33^ ^,^ ^34^. Although the use of the Pringle Maneuver seems to decrease intra-operative bleeding as a result of portal vein and hepatic artery occlusion, it may also increase the risk of acidosis caused by liver reperfusion. However, we did not observe an impact on surgical or oncologic outcomes^34^. Sanjay *et al*.^35^ reported a systematic review with four RCTs comparing transection with and without inflow pedicle occlusion and showed a shorter transection time in the group with Pringle maneuver, although with no significant differences in blood loss, transfusion rates, morbidity or mortality. Our preference is to use the Pringle maneuver selectively for patients with a higher risk of bleeding during liver transection, as in patients with steatosis or sinusoidal dilation, but always in an intermittent way and for the shortest duration possible, to avoid putative colon congestion during colectomy, which might compromise the quality of the anastomoses, as suggested in animal models^36^. Moreover, patients should have a good performance status and be fit to undergo a simultaneous resection taking into account the need for pneumoperitoneum and the risk of acute bleeding and postoperative complications. Therefore, as to the technical aspects including anesthesiology and surgical planning, it seems to be safer and easier to perform the liver resection first since we can offer low CVP and Pringle maneuver without jeopardizing intestinal anastomosis, which will be performed later in the volume-recovering phase after hepatectomy (to improve renal function and intestinal perfusion). Thus, after Pringle maneuver venous congestion decreases and the arterial perfusion is optimized by a higher CVP as a result of volume recovery after hepatectomy. The colonic anastomosis could be performed later with a lesser risk after hepatectomy, avoiding congestion and low perfusion.

## Conclusions

The simultaneous resection of CRC and CRLM seems to be safe when patients are carefully selected. However, there are no RCTs supporting it. The presence of the primary tumor and its symptoms can direct the treatment to a primary first approach (resection and /or deviation), and then chemotherapy. For asymptomatic patients or patients whose primary tumor has been treated, the liver should be approached according to risk of recurrence: if there is a low-risk of recurrence, it could be approached first; otherwise, it should undergo chemotherapy first. To identify “good responders” and avoid early recurrence after simultaneous resections is a patient selection tool since synchronous CRLM per se represents an important prognostic factor for recurrence suggesting systemic disease and claims for pre-operative chemotherapy. The association of colorectal and liver surgeons for the surgical planning of simultaneous resection is mandatory, including trocar position, procedure sequencing, and patient position.
